# Essential readings in international and comparative adult education: Jost Reischmann (Ed.). Ziel Verlag, Augsburg, 2021, 320 pp. ISBN 978-3-96557-093-1 (pbk)

**DOI:** 10.1007/s11159-022-09951-z

**Published:** 2022-05-02

**Authors:** Bernd Käpplinger

**Affiliations:** Justus-Liebig-University Giessen, Giessen, Germany

With this book, Jost Reischmann presents an impressive collection that has the potential to become a “must-have” for people who deal with adult education in an international context. The emeritus professor from Bamberg (Germany) has compiled key texts covering more than 100 years of comparative and international adult education research, ranging from its early forms to the present. The earliest item in the collection is from 1900 by Michael E. Sadler from Britain; it addresses the still pertinent question of what we can learn for our own practice from studying foreign education systems. Sadler’s text demonstrates that an international perspective already existed at the beginning of the professionalisation of the field of adult education. One of the most recent texts, co-authored by Qi Sun (USA/China) and Elizabeth Erichsen (USA) is from 2012. They raise the question of how to bridge the Western and Eastern worlds of adult education – a question perhaps even more relevant and important than ever in these war-ridden times.

The colourful spectrum of 30 seminal texts is presented in eight sub-sections, spanning the following categories: history/development; travellers and their reports; international/programme reports/country reports/juxtaposition; comparative studies; international organisations and institutions; lessons learned; epilogue 2000–2020; and observations/perspectives. Each of the items in the collection is preceded by an introduction of the respective author(s) and a few editorial remarks. These introductions help the reader access the texts, although many of the featured authors are well-known to scholars in the field. Some readers might have wished for the editorial remarks to be longer, since they are quite insightful. The value of this book should not be underestimated, because to my knowledge this is the first time that classic texts of the field of international and comparative adult education are brought together in one volume. In this respect, this anthology will be a valuable resource for teaching.

One criticism, however, is that there are too many White, Western, English-speaking men represented in this volume. To be fair, Reischmann himself addresses this issue by pointing out that the compilation reflects how international and comparative adult education research was visibly set up decades ago. Here it would take more in-depth historical research and effort to make significant contributions by women or non-Western countries more visible. Thus, the whole volume can also be understood as an invitation to dig deeper and search for perhaps lost or hidden traces.

The significant increase of contributions by women among the more recent texts greatly enriches the book. In this regard, I would like to point out a very interesting article by the Serbian scholar Katarina Popovic at the end of the volume. However, equal gender representation is still far from being achieved, as is equal representation of the Global North, Global South, Global West and Global East. There is still a lot of work to do for the field in order to become truly international beyond anglophone international.

At the end of the more than 300 pages of the volume, Jost Reischmann offers his own observations and perspectives. He also critically reflects on how the texts were collected for this publication. His honest and critical discussion of the limitations of the book will inspire future generations of authors who want to engage in a similarly bold endeavour. Information is also provided on the current state of international comparative research and where the journey of the field might lead in future. While he does not idealise the situation, he provides a rather critical inventory.

After two pandemic COVID-19 years with many travel restrictions and even closed borders, I was struck by the observations of how important travel is for international comparative research and what role international friendships have played in both the past and the present for everyone involved. In this respect, the articles and the entire volume do not represent a sober, distanced analysis of a cold-blooded scholar, but a committed and humane plea for an international dimension in adult and continuing education that goes beyond competition and country comparisons à la PISA. For example, some of the contributions are related to peace education, an issue many of us have lost sight of in light of the prominence of economic development. Overall, the personal engagement and empathy of the editor Jost Reischmann comes through very strongly.

It will be exciting to see how comparative and international adult education research and practice will continue to develop over the next few decades. The achievements of previous generations should not be intimidating, although they are impressive, as this volume demonstrates. This book is highly recommended to readers who are interested in the history of the field of adult education and/or who wish to get an overview of the key contemporary issues in the field and want to do international research. This volume constitutes a truly essential contribution, although the history and histories of the field are certainly even more pluralistic than the volume has been able to capture. The present and the next generations of researchers should build on this valuable book and take it as an invitation to shed more light on other houses and rooms not known to all of us yet.

